# Pencilbeam Irradiation Technique for Whole Brain Radiotherapy: Technical and Biological Challenges in a Small Animal Model

**DOI:** 10.1371/journal.pone.0054960

**Published:** 2013-01-28

**Authors:** Elisabeth Schültke, Michael Trippel, Elke Bräuer-Krisch, Michel Renier, Stefan Bartzsch, Herwig Requardt, Máté D. Döbrössy, Guido Nikkhah

**Affiliations:** 1 Division of Stereotactic and Functional Neurosurgery, Freiburg University Medical Center, Freiburg, Germany; 2 European Synchrotron Radiation Facility (ESRF), Grenoble, France; 3 Deutsches Krebsforschungszentrum (DKFZ), Heidelberg, Germany; Dresden University of Technology, Germany

## Abstract

We have conducted the first *in-vivo* experiments in pencilbeam irradiation, a new synchrotron radiation technique based on the principle of microbeam irradiation, a concept of spatially fractionated high-dose irradiation. In an animal model of adult C57 BL/6J mice we have determined technical and physiological limitations with the present technical setup of the technique. Fifty-eight animals were distributed in eleven experimental groups, ten groups receiving whole brain radiotherapy with arrays of 50 µm wide beams. We have tested peak doses ranging between 172 Gy and 2,298 Gy at 3 mm depth. Animals in five groups received whole brain radiotherapy with a center-to-center (ctc) distance of 200 µm and a peak-to-valley ratio (PVDR) of ∼ 100, in the other five groups the ctc was 400 µm (PVDR ∼ 400). Motor and memory abilities were assessed during a six months observation period following irradiation. The lower dose limit, determined by the technical equipment, was at 172 Gy. The LD50 was about 1,164 Gy for a ctc of 200 µm and higher than 2,298 Gy for a ctc of 400 µm. Age-dependent loss in motor and memory performance was seen in all groups. Better overall performance (close to that of healthy controls) was seen in the groups irradiated with a ctc of 400 µm.

## Introduction

The high photon flux of synchrotron-generated X-rays allows the design of radiotherapeutic approaches that could not be executed with the radiotherapy equipment presently available in the hospital environment. Microbeam radiation therapy (MRT) is a synchrotron-based experimental concept of spatially fractionated radiotherapy. In one single MRT irradiation session it is possible to apply radiation doses that are higher by at least one order of magnitude, compared to the total radiation dose commonly applied during a complete cycle of temporally fractionated radiotherapy for malignant brain tumors in the hospital setting. Characteristic for microbeam radiotherapy is the application of a spatially fractionated high dose of X-rays generated by a special collimator [1]. It has been shown that the overall structure of the healthy tissue between the paths of the microbeams stays intact [2] and that the function of healthy tissue in the path of the beam is greatly preserved after MRT, possibly taking over part of the function of the tissue destroyed in the path of the microbeams [3], [4]. The application of high irradiation doses should prove an advantage in the work with highly radioresistant tumors. It has been shown previously that microbeam radiation therapy significantly increases survival time in animal models of malignant brain tumor [2], [3], [5], [6], [7], [8]. At the European Synchrotron Radiation Facility (ESRF) in Grenoble (France) the technical equipment for pencilbeam irradiation, a new concept based on the principles of microbeam irradiation, has been recently developed. The three-dimensional distribution of high-dose X-ray deposits suggests that the tissue tolerance for pencil beams should be superior when compared to the original microbeam irradiation. Thus, the tolerance of healthy brain tissue to pencilbeam irradiation might be sufficiently high to use pencilbeam irradiation for whole brain radiotherapy (WBRT). We have therefore conducted a first series of *in-vivo* experiments using pencilbeam irradiation in healthy adult mice, to define upper and lower dose limits dictated by the physiological reaction of our experimental animals as well as by the technical equipment.

## Materials and Methods

### 2.1 Animal Model and Ethics Statement

All animals were housed and cared for in a temperature-regulated animal facility exposed to a 12-hr light/dark cycle. All experiments were performed in accordance with the guidelines of the French and German Councils on Animal Care. The study was specifically approved by the Institutional Animal Care and Use Committees of the participating institutions (Freiburg University Medical Center and ESRF).

Since one of the experimental goals was a behavioural post-irradiation assessment, we have used adult healthy C57 BL/6J mice (Charles River France, 27.5–32.5 g) for our experiments, one of the most suitable small animal species for behavioural testing. Out of 58 animals, fifty animals underwent pencilbeam irradiation, distributed into ten groups (n = 5/group), the remaining eight animals served as non-irradiated controls (Table 1).

**Table 1:** Overviev over the parameters and number of animals used in each of the experimental groups.

Peak dose	172 Gy	431 Gy	459 Gy	862 Gy	1,146 Gy	1,164 Gy	1,724 Gy	2,298 Gy
**ctc**								
200 µm	5	5		5		5	5	
400 µm			5	5	5		5	5

The irradiation experiments were conducted under general anaesthesia, induced by 1.5–2% Isoflurane® in oxygen inhalation and upheld by an intraperitoneal injection of a Ketamine and Xylazine cocktail (Ketamine 1 mg/10 g, Xylazine 0.1 mg/10 g). After the radiotherapy, the animals were allowed to recover. The irradiation study was followed by a six months observation period, with monthly tests for motor abilities and the ability to form new memory contents.

### 2.2 Monte Carlo Calculations of the Peak-to-valley Dose Ratio (PVDR)

Monte Carlo simulations were carried out in the Geant4 tool set (Version 4.9.3 p02) using an adjoint simulation technique. As target a cube of water was defined with 16 cm side length. Energy was scored in 5×5 µm^2^ voxels with a height of 4 mm. At the reference depth of 3 mm the height was reduced to 0.5 mm in an additional simulation, since the dose dependency at shallow penetration depth is steep. In this depth Monte Carlo simulations were compared to film dosimetry. In adjoint Monte Carlo simulations source and detector geometry can be inverted. This can help to reduce calculation time and reduce statistical uncertainties. In this case the lateral geometry perpendicular to the beam was inverted. The source was modulated as a point source emitting a parallel horizontally polarised beam with the ESRF spectrum [9]. The beam was set linearly polarised and the Livermore libraries for electromagnetic interactions at low energies were used, that include interaction cross sections of polarised particles. The detector has the lateral shape of the collimator in the real world and is represented by the corresponding combination of scoring bins. As long as lateral inhomogeneities in the absorber material can be neglected, lateral dose distributions can be obtained by shifting the scoring bins perpendicular to the beam. This is the case for a homogeneous water phantom of the given size. The uncertainty of the method based on this model should be small than 2%.

### 2.3 Measurement of the PVDR

The calculated PVDRs were verified by dose measurements using Gafchromic film (HD-810) [10], read out 48 hours after irradiation with a microdensitometer (JL Automation) [11]. A solid water phantom RW3 (Goettingen White Water) was used with individual slab dimensions of 30 cm × 30 cm. The overall thickness of the plates corresponded exactly to 15 mm in depth equivalent to the depth of a mouse brain. The solid water slabs were composed of one 1 mm, two times 2 mm and one 10 mm plate. Films were placed at an equivalent depth of 1, 3 and 5 mm at the edge of the upper right corner to irradiate an 18 mm wide and 8 mm high field corresponding to the one used for the mouse irradiations. In this geometry, the equivalent thickness of a mouse brain was provided as well as the little scattering contributions from the right and the top directions. There was, however, substantially more scattering from the left and bottom areas of the plates, due to the non-availability of a perfectly suitable solid water phantom identical to the anatomical dimensions of a mouse. For this reason the measured valley dose should correspond to an upper limit and subsequently the measured PVDR should be lower than the Monte Carlo calculated PVDR.

The quantitative analysis of the films was based on the direct comparison in optical density from a series of calibrated films of known dose values. The films were irradiated from the same sheet of Gafchromic film with a homogenous field of 2 cm×2 cm in 2 cm depth previously measured with a calibrated ionization chamber (PTW 31010) [12], [13] and cross-checked with a second set of calibration films exposed at a conventional X-ray generator. Due to the non-uniformity of the Gafchromic films and the technical challenges inherent in the use of the microdensitometer, errors as high as 10% are reasonable to be quoted for absolute dose measurements using this technique.

### 2.4 Pencilbeam Irradiation

**Figure 1:**
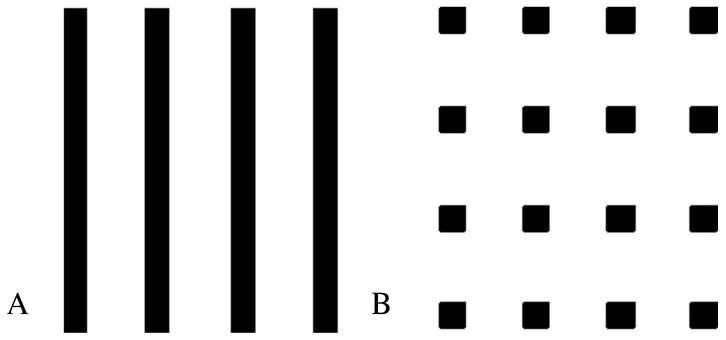
Schematic comparison between the profile of energy deposition in the direction of beam propagation with microplanar beams as in the classic MRT approach (A) and in pencilbeam irradiation (B). Based on the geometry of beam deposition we would expect a higher tolerance of high dose irradiation in the normal tissue for the pencilbeam approach (less interruption of normal structure).

The irradiation experiments were conducted at ID 17, the biomedical beamline of the European Synchrotron Radiation Facility (ESRF) in Grenoble. With the insertion of a multislit collimator (MSC) [1] and a step and shoot method, square pencilbeams can be produced at ID 17. The vertical polished tungsten carbide slits provide a beam height of 50 µm, and the single stack MSC will shape the beam spots to 50 µm by 50 µm square pencil beams with 400 center-to-center (ctc) distance for the desired total width of 18 mm resulting in 45 pencilbeams per line. For the 200 ctc irradiations first a lateral shift of 200 µm is performed prior to a displacement in Z, while for the 400 ctc spacing, only 20 displacements of 400 µm each in height are required for the 8 mm high field at typical exposure times between 0.01 and 0.2 seconds. The entire irradiation time is relatively short for small volumes, where the surface to be covered by the beam array measures only a few millimetres. For the administration of whole brain irradiation, the valley dose should be minimal yet a therapeutically efficient peak dose must be administered. Looking at the three-dimensional geometry of the beams within a given tissue volume, we expect that the normal tissue tolerance of pencilbeams should be higher than for microplanar beams. With the microplanar beams of the classic microbeam irradiation approach, the brain is radiosurgically sectioned into parallel slices. The pencilbeam approach, on the other hand, is characterized by the deposition of small high-dose deposits that avoid the radiosurgical slicing effect (Figure 1). As a result, we expect a better repair capacity in the healthy brain tissue.

**Figure 2:**
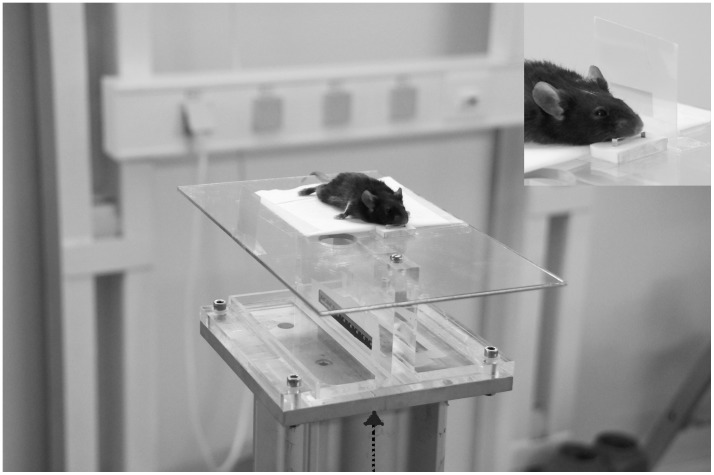
Experimental setup for pencilbeam irradiation. The animal was positioned orthogonally to the direction of beam propagation (right to left) and dose delivery was verified using Gafchromic film, visible in right lateral position. For pencilbeam irradiation, the goniometer with the animal was moved vertically through the beam, along the axis marked by the broken arrow.

White synchrotron radiation X-ray beam, delivered from a wiggler source, was filtered through 1 mm copper and 1.5 mm aluminum, the energy spectrum extending from about 50 keV to well above 350 keV with a maximum at 83 keV [14]. We used quasi-parallel microbeams of 50 µm width in a step-and-shoot mode to create a field size that covered the entire mouse head for whole brain irradiation. The mice were positioned prone on the goniometer, with the top of the skull horizontal and their teeth hooked into a small fixed holding device. Irradiation was administered in unidirectional mode and in right-to-left lateral direction, with the animals positioned orthogonally to the direction of beam propagation (Figure 2). The goniometer with the animal was moved vertically through the beam, stopped for the irradiation and then advanced further in vertical direction until the entire head was covered in the irradiation field. Keeping the beam width constant at 50 µm, we tested two different ctc distances, where the distances between the centres of two neighbouring parallel beams were either 200 µm or 400 µm. We tested peak doses ranging between 172 and 1,724 Gy for ctc 200 µm and between 459 Gy and 2,298 Gy for ctc 400 µm at 3 mm depth, the assumed centre of the implanted tumour. The entire radiation dose was administered in one single session, lasting between 2 and 6 minutes, depending on the ctc and the dose administered. Gafchromic film [13] was used in lateral position to document irradiation geometry and irradiation dose.

### 2.5 Behavioural Testing

The impact of the irradiation process on the animals’ motor and cognitive abilities was assessed. Rather than administering an exhaustive test battery, we focused on two frequently repeated behavioural tasks which have an important equivalent in the assessment of the quality of life for human patients: the rotarod test as assessment for motor abilities, coordination and endurance, and an object recognition test for the assessment of new memory formation.

Assessment of motor abilities (rotarod test): The rotarod test is a widely used test providing a simple, drug-free evaluation of overall motor deficits in rodents. The apparatus used consists of a central horizontal rod, separated into five compartments by circular dividers, and driven to rotate by an attached belt controlled by a programmable motor (Ugo Basile, Comerio, VA, Italy). Mice, habituated to the apparatus 24 h prior to testing, were placed, facing away from the investigator, onto the central horizontal axis of the rotarod with the axis rotating at 40 rpm. Each animal had 5 trials to remain on the rotating axis as long as possible, with a maximum trial time of 240 seconds. The best performance of each individual animal was used to calculate group averages.

#### Assessment of cognitive abilities (Object Recognition Test)

Rodents show a strong tendency to approach and explore novel rather than familiar objects. This feature of cognitive behaviour, related to the animals’ ability to form new memory contents, was exploited in the current study by using the Object Recognition Test (ORT) developed by Ennaceur and Delacour [15].

Animals were habituated during a 4 min single session to the empty test environment which was a 40 cm×40 cm×60 cm open black Perspex box with a light grey floor. The following day, animals were returned to the test environment where two identical objects had been placed on the floor (T1), and allowed to explore the objects for 4 min before returning to their home cage. Confronted with this scenario, animals will typically spend equal times exploring each of the objects. Retrieved from their cage after a 4 hour inter-trial interval, animals were tested again (T2), but for this session one of the already familiar objects was replaced by a new object with similar salience. The time spent exploring the objects was again measured. This test is based on the hypothesis that mice with normal memory function will spend more time examining the new, rather than re-exploring the previously encountered object. On the other hand, animals with memory encoding or retrieving impairments will again spend equal times with both objects.

The Rotarod and the Object Recognition tests were conducted over a period of 6 months in the behavioural laboratories of the Division of Stereotactic Neurosurgery in Freiburg. Both tests were conducted at two and four weeks after irradiation and at monthly intervals thereafter for 6 months. The first rotarod test was first conducted one week after irradiation.

### 2.6 Histology

At the end of the 6 months observation period, the animals were sacrificed by an overdose of Ketamine and the head was removed. The brains were cut either sagittally along the midline or axially through the olfactory bulbs and the cerebellum, placed into histology cassettes and stored in 10% phosphate-buffered formalin for five days. After embedding in paraffin, 5 µm thick sections were mounted on glass slides and stained according to a standard H&E protocol.

## Results

### 3.1 LD50 for Animals Irradiated at ctc 200 µm

Within the first 24 hrs after irradiation, four out of 5 animals died in the ctc 200 µm group that had received a peak dose of 1,724 Gy and a valley dose of 17,24 Gy. Also within the first 24 hrs after irradiation, two out of five animals died in the ctc 200 µm groups after receiving a peak dose of 1,164 Gy and a valley dose of 11.64 Gy. Therefore, the LD50 for a ctc of 200 µm lies around the peak dose of 1,164 Gy, possibly slightly above that value. There were no further unexpected deaths beyond 24 hrs after irradiation. All other animals survived the observation period of six months after irradiation. The decision to terminate the experiment was taken six months after irradiation once the age-dependent performance loss had reached 33% in the healthy control animals.

### 3.2 Peak-to-valley Dose Ratios

**Figure 3:**
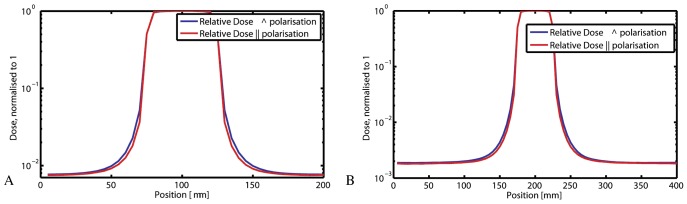
Beam profiles for ctc 200 µm (A) and ctc 400 µm (B) at 3 mm depth. Profiles parallel and perpendicular to the polarisation vector are shown. The FWHM is 55 µm.

**Figure 4:**
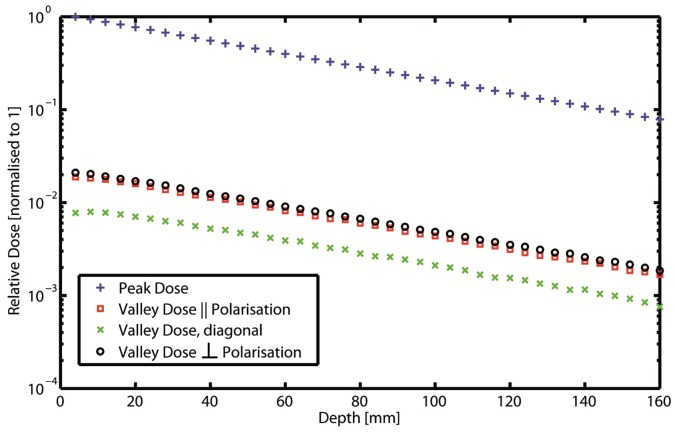
Peak and Valley doses in dependency on depth in the centre of the pencil beam field. The irradiation field contains 45×21 pencil beams and the phantom is approximated as a homogeneous box of water with 8 cm side length. Valley dose can be measured vertically or laterally between two pencil beams and diagonal in the centre between two beams. The latter gives the lowest value due to the greatest distance from the beams. The other two valley doses differ slightly due to polarisation effects.

According to Monte Carlo calculations for 18 mm×8 mm fields the PVDRs for 50 µm×50 µm square pencil beams were expected to be about 125 for a ctc of 200 µm and about 530 for a ctc of 400 µm. The beam profiles are shown in Figure 3, while Figure 4 illustrates the steep incline in the PVDR-curve with depth. The use of Gafchromic film proved very useful for the documentation and verification of the administered radiation geometries in our small animal model. With PVDR values of about 100 for a ctc of 200 µm and about 400 for a ctc of 400 µm, the measured PVDRs were slightly lower than the calculated values. This confirmed our expectation that the measured PVDRs would be lower than the calculated PVDR, due to the imperfection of the model used for the measurements.

For further reference in this paper, we will work with the measured (i.e. lower) PVDRs for safety reasons.

### 3.4 Behavioural Testing

**Figure 5:**
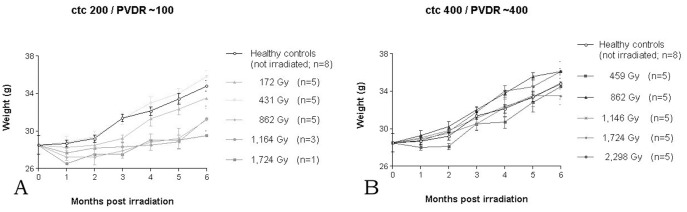
Weight curves for the animals in the two beam configurations tested, ctc 200 µm (A) and ctc 400 µm (B). The irradiation doses in the legends are given as peak dose.

There was an initial light to moderate weight loss in the first month after irradiation in all but one of the five ctc 200 µm groups, but in only one out of five ctc 400 µm groups (Figure 5). The most dramatic weight loss (about 10% of body weight) was seen in the one surviving animal that had received a peak dose of 1,724 Gy with a ctc of 200 µm. The average weights stayed significantly below that of the healthy controls in three out of five groups irradiated with a ctc of 200 µm for the duration of the six months observation period, while in the five ctc 400 µm groups all animals had regained their weights between the second and third month after irradiation; in two of those groups, the average weights were slightly above that of the healthy controls.

**Figure 6:**
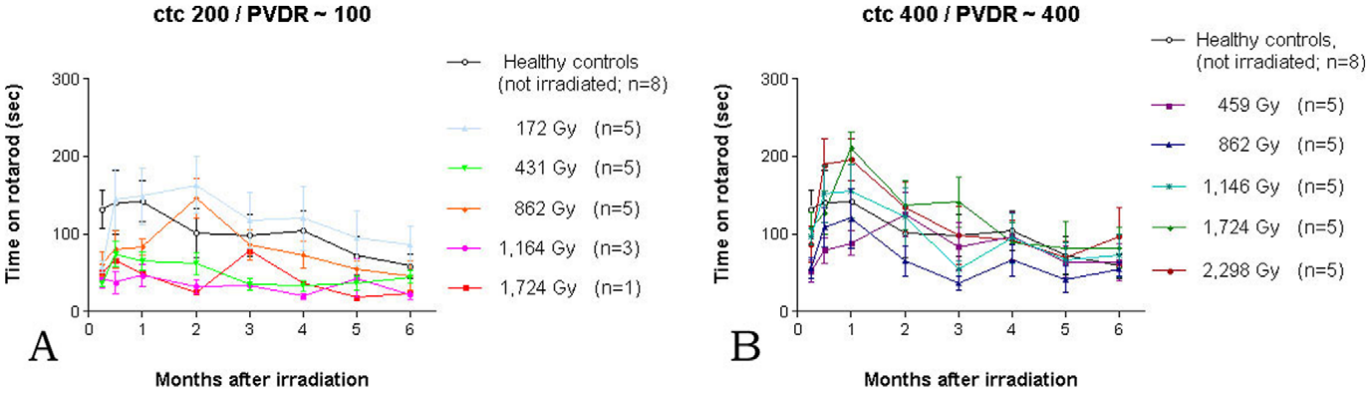
Duration of performance on the rotarod over the 6 months observation period. Performance was generally better in the ctc 400 µm groups with a PVDR of ∼ 400 (B), compared to the animals in the ctc 200 µm groups with a PVDR of ∼ 100 (A).

Neither motor nor memory performance appeared to be closely correlated to the weight development. An overall better performance was seen in the ctc 400 µm groups (PVDR ∼ 400), compared to the animals in the ctc 200 µm groups (PVDR ∼100). A slow decline of motor performance was seen starting at about 2 months into the observation period in the healthy controls and in the irradiated animals (Figure 6), most likely age-dependent. For the five ctc 400 µm groups with peak doses between 352 Gy and 1767 Gy, the average group performance was close to that of healthy controls in four of the groups. For the five ctc 200 µm groups with peak doses between 172 Gy and 1,724 Gy, only the animals that had received a peak dose of 172 Gy performed close to the healthy controls. In two groups (peak doses of 431 Gy and 862 Gy) the motor performance recovered to within 25% of that seen in healthy controls. The performances of the one remaining animal in the 1,724 Gy group and the three remaining animals in the 1,164 Gy group were stable at low level (less than 40% of healthy controls). Thus, we observed a dose-dependence of motor performance within the ctc 200 µm groups.

**Figure 7:**
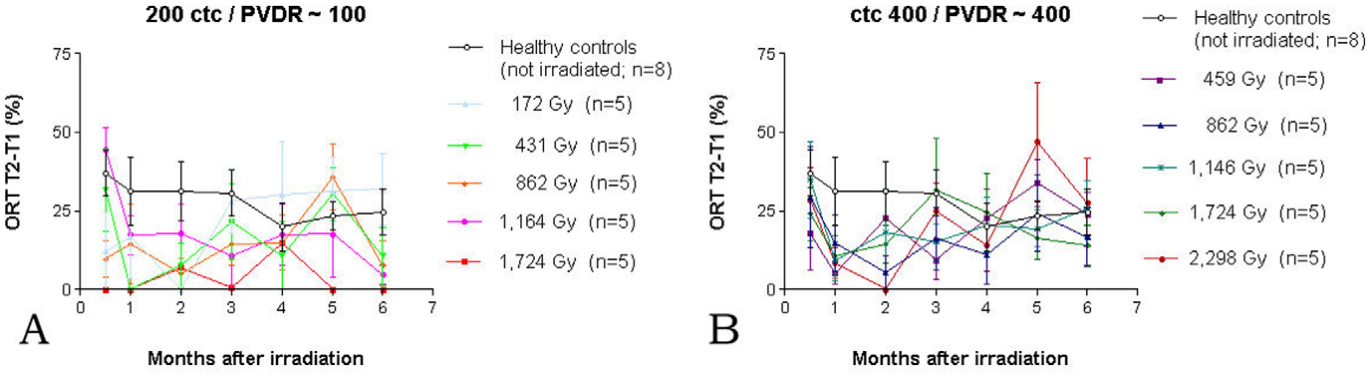
Ability to form new memory as assessed by Object Recognition Test (ORT). Performance was generally better in the ctc 400 µm groups (B), compared to the animals in the ctc 200 µm groups (A). The initial drop of memory performance is lagging behind that of motor performance by several weeks.

Similar to motor the performance, also the overall memory performance was better in the ctc 400 µm groups, compared to the animals in the ctc 200 µm groups. The age-dependent performance drop with memory performance was not as noticeable as with the motor performance (33% vs. 58.6% for the healthy controls). However, a post-irradiation memory performance drop was seen in all groups of irradiated animals, with the lowest performance level being reached between one and three months after irradiation (Figure 7).

Out of the five ctc 200 µm groups irradiated with peak doses between 172 Gy and 1,724 Gy, only in the 172 Gy group did memory performance recover to the level of healthy controls. The one surviving animal from the ctc 200 µm group after a peak dose of 1,724 Gy performed very poorly throughout, while the three surviving animals of the 1,164 Gy group performed surprisingly well at 2 weeks after irradiation, while thereafter their performance level dropped to about 35% of that seen in healthy controls. A performance drop significantly below the level of healthy controls was seen at six months after irradiation in three others of the ctc 200 µm groups. Only the animals in the 172 Gy group performed continuously close to healthy level.

In three out of five ctc 400 µm groups did the motor performance reach its lowest level one month after irradiation, followed by recovery to within 57% and 67% in the worst performance groups (1,724 Gy and 862 Gy peak dose, respectively) and performance equal to or even slightly better than the healthy controls (459 Gy, 862 Gy and 2,298 Gy peak dose). Thus, no clear dose-dependence could be established for memory performance in the ctc 400 µm groups. After irradiation, the initial drop of memory performance was lagging behind the initial drop of motor performance by several weeks.

### 3.5 Histology

**Figure 8:**
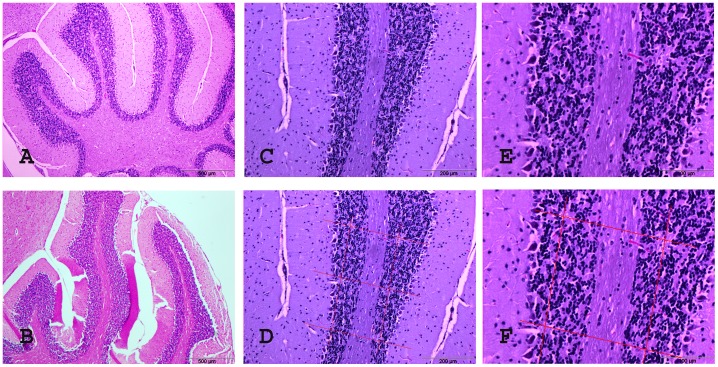
H&E histology, sagittal sections of the cerebellum 6 months after irradiation. C57 BL/J6 mice, 6 months after irradiation with a peak dose of 172 Gy/valley dose of 1.72 Gy, ctc 200 µm (A, C-F are enlargements from sample A) and healthy control (B). The lighter spots almost devoid of cells correspond to the ctc 200 µm lateral Gafchromic film profile as can be demonstrated by the overlaid patterns of histology section and distance grid with 200 µm lateral dimensions (D and F). Because the geometry of mounted tissue sections is influenced by several aspects of the preparation process, the histological pattern does not perfectly correspond to the pattern recorded on Gafchromic film (i.e. the grid lines are not quite orthogonal).

The geometry of the irradiation field is reflected very well in the post-mortem histology, especially in the cell-dense layers of the cerebellum (Figure 8). The brain shown in Figure 8 received whole brain irradiation with a ctc of 200 µm and the animal survived the full observation period of 6 months after irradiation. In the sagittal sections of the brain we can very well distinguish the pattern of spots almost devoid of functional cells, corresponding to the geometric grid of the pencil beam irradiation caused by the right-to-left lateral irradiation with a ctc of 200 µm. Even despite some growth of the animals during the 6 months observation time and some shrinkage of the tissue during the paraffin embedding process, the distances between the centres of the spots correspond to the ctc of 200 µm.

**Figure 9:**
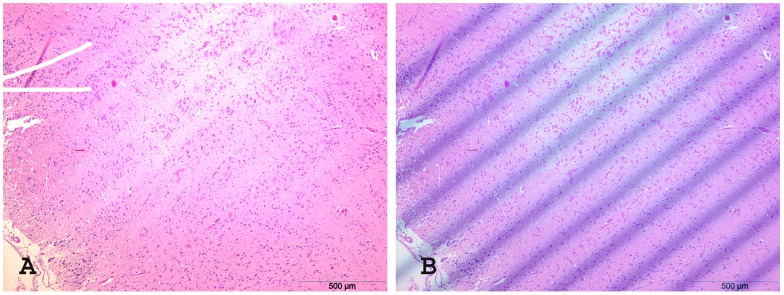
H&E histology of C57 BL/J6 mice, axial sections 6 months after irradiation. Showing a 5 µm thick axial section after irradiation with a peak dose of 1,164 Gy and a valley dose 11.64 Gy, ctc 200 µm (A) and the axial histology section overlaid with the photo of the lateral beam profile (B). Only a few cells, some of them with small dark (pyknotic) nuclei, are left in the path of the beam.

Another cell-dense structure in the brain is the hippocampus. Although it is technically challenging to cut microtome sections exactly within the plane of a microbeam, it is possible to see the stripes almost devoid of cells caused by the pencilbeams, running through part of the plane in the axial microscopic section (Figure 9). The overlay with the irradiation pattern recorded on Gafchromic film (Figure 9b) verifies again the concordance with the irradiation pattern of 200 µm. Like in Figure 8, we can also see in this axial microscopic section some small dark pyknotic nuclei likely destined for apoptotic cell death. Considering that the geometry of mounted tissue sections is influenced by fixation and embedding processes as well as by the mounting process, the microphotographs represent well the expected distribution of the irradiation pattern expected based on irradiation planning and recording on Gafchromic film.

## Discussion

First of all, we have shown in our experiments that it is technically possible to conduct pencilbeam irradiation *in-vivo* in a small animal model. The results of the histological analysis suggest that even with our technically very simple step-and-shoot approach and the resulting longer duration of the irradiation procedure, compared to the smooth continuous movement of the animal through the beam as known from classic microbeam radiation therapy, a fairly precise three-dimensional irradiation pattern can be achieved. However, a technical solution can easily be implemented to cope with larger brains and/or with more complicated irradiation geometries, by fast scanning method to reduce the irradiation time in order to avoid smearing of the beam paths due to the pulsation of the brain. Therefore, if one could show in a follow-up experiment that pencilbeam irradiation is therapeutically efficient within the limits set by the present technical equipment and by the adverse effects of the radiation (inacceptable decline of motor and memory performance), it would be worth to implement the technical equipment to allow a continuous movement of the animal through the beam, whereby a grid system between source and target would ensure the specific pencilbeam geometry. This would decrease the total duration of the irradiation process and give pencilbeam irradiation the same advantage as classic microbeam radiation therapy: the ability to deposit high X-ray doses in an extremely short time, so that the possible smearing of beam edges because of brain pulsation should cause no serious concern.

Patients with multiple brain metastases present a challenge to therapeutic planning. Whole brain radiotherapy (WBRT) is often the only available therapeutic option, because many drugs used commonly to curb metastatic growth in other organs do not pass the blood-brain barrier. One of the most commonly reported morphological problems after WBRT are longterm changes in the white matter, resulting in behavioural changes and cognitive deficits [16], [17], [18], [19], [20], [21]. The structural damage caused by the radiosurgical transection of the pencilbeams, visible in the histology of the hippocampus in our samples, would suggest that behavioural changes might be caused also with pencilbeam irradiation, depending on beam width, three-dimensional beam geometry and irradiation doses. For this reason, we choose to test both motor and memory function after irradiation in our initial set of pencilbeam experiments.

Based on the three-dimensional beam geometry of the irradiation field and the fact that there is less radiosurgical dissection of healthy brain tissue with pencilbeam as compared to the classic microbeam irradiation, we can expect healthy brain tissue to be more tolerant to pencilbeam irradiation than to either broad beam irradiation or classic microbeam irradiation. In fact, we have already shown in our small animal model that irradiation doses higher than 800 Gy can be administered with a ctc of 400 µm in one single fraction of whole brain irradiation, with performance recovery comparable to that of healthy controls. While an initial drop in motor and memory performance was seen in all groups after irradiation, there was fairly good recovery in most of the groups receiving irradiation with a wider ctc (400 µm vs. 200 µm) and a higher PVDR (400 vs. 100). Considering that the average life of laboratory mice is between one and two years, our six months observation period after irradion correlates to 25–50% of the animals’ life span. That at an equal beam width both motor and memory performance were better with a larger ctc (400 µm vs. 200 µm) confirms the observations made by other authors, that smaller spacings between the beams causes higher toxicity on the brain [8]. Interestingly, other authors also found that when treating brain tumours in an animal model, the rate of cure was higher with larger compared to more narrow beam spacings where equal irradiation doses were applied [22]. The lower rate of adverse effects provides a further supporting argument to favour future pencilbeam experiments with a ctc of 400 µm rather than 200 µm.

### Conclusion

Pencilbeam irradiation of the whole brain in a small animal model is technically feasible and well tolerated even at high peak doses. The next step is to investigate whether we can achieve tumour control with either of the beam configurations within the limits determined in this set of experiments.
